# 3E-Net: Entropy-Based Elastic Ensemble of Deep Convolutional Neural Networks for Grading of Invasive Breast Carcinoma Histopathological Microscopic Images

**DOI:** 10.3390/e23050620

**Published:** 2021-05-16

**Authors:** Zakaria Senousy, Mohammed M. Abdelsamea, Mona Mostafa Mohamed, Mohamed Medhat Gaber

**Affiliations:** 1 School of Computing and Digital Technology, Birmingham City University, Birmingham B4 7AP, UK; zakaria.senousy@mail.bcu.ac.uk (Z.S.); mohamed.gaber@bcu.ac.uk (M.M.G.); 2 Faculty of Computers and Information, Assiut University, Assiut 71515, Egypt; 3 Department of Zoology, Faculty of Science, Cairo University, Giza 12613, Egypt; mmostafa@gu.edu.eg; 4 Faculty of Basic Sciences, Galala University, Suez 435611, Egypt; 5 Faculty of Computer Science and Engineering, Galala University, Suez 435611, Egypt

**Keywords:** breast cancer, histopathological images, entropy, uncertainty quantification, elastic ensemble, dynamic ensemble, convolutional neural networks

## Abstract

Automated grading systems using deep convolution neural networks (DCNNs) have proven their capability and potential to distinguish between different breast cancer grades using digitized histopathological images. In digital breast pathology, it is vital to measure how confident a DCNN is in grading using a machine-confidence metric, especially with the presence of major computer vision challenging problems such as the high visual variability of the images. Such a quantitative metric can be employed not only to improve the robustness of automated systems, but also to assist medical professionals in identifying complex cases. In this paper, we propose Entropy-based Elastic Ensemble of DCNN models (3E-Net) for grading invasive breast carcinoma microscopy images which provides an initial stage of explainability (using an uncertainty-aware mechanism adopting entropy). Our proposed model has been designed in a way to (1) exclude images that are less sensitive and highly uncertain to our ensemble model and (2) dynamically grade the non-excluded images using the certain models in the ensemble architecture. We evaluated two variations of 3E-Net on an invasive breast carcinoma dataset and we achieved grading accuracy of 96.15% and 99.50%.

## 1. Introduction

Breast cancer is a major public health concern around the world, where its prevalence rate is the second-highest rate for women (excluding lung cancer) among all forms of cancer [1]. The study of histopathological images remains the most commonly used tool for diagnosing and grading breast cancer, even with the substantial advances in medical science. Early diagnosis can dramatically improve the effectiveness of therapy. The symptoms and signs of breast cancer are numerous, and the diagnosis encompasses physical analysis, mammography, and confirmed by core needle biopsy tissue (CNB) from the suspicious breast area. The sample tissue extracted from the CNB process demonstrates the cancerous cells and the grade of cancer associated with them. Pathologists typically look for certain characteristics that can help them predict disease prognosis during the visual inspection of the biopsy specimen of the tissue (i.e., what is the likelihood of cancer spreading and growing?).

For tumor grading, pathologists usually use the Nottingham scoring system that depends on morphological changes including glandular/tubular formation, nuclear pleomorphism, and mitotic count [2]. Due to the high visual variability of the samples in terms of their morphological structure, visual qualitative grading assessment is a time-consuming and laborious process [3]. In the context of histopathological image analysis, grading of invasive breast cancer provides many challenging problems. First, there are variations in subjective criterion evaluation between observers when it comes to diagnosis/grading. Second, it is difficult to capture the proper combination of features and the morphological heterogeneity within the tumor regions [3,4]. Such challenges usually lead to substantial effort and exhaustive manual qualitative study from pathologists. Thanks to computational pathology which helped in alleviating this burden in recent years. In computational pathology, deep learning (DL) approaches have made tremendous progress and achieved outstanding results, leading many researchers to provide automated and unbiased solutions for several different histopathological image analysis applications including breast cancer grading and tissue classification [5]. Deep convolution neural networks (DCNNs) are the most commonly used type of DL approaches, demonstrating outstanding performance in extracting image salient features for the different computational pathology applications [6].

Despite the prevalence of DCNNs in several histology image analysis applications including grading, the ability of a single DCNN model to obtain discriminatory features is constrained and usually results in sub-optimal solutions [7,8,9]. As a consequence, an ensemble of DCNN models has been proposed to conserve the description of histopathological images from recognizable perspectives to a more precise grading [10]. More importantly, to the best of our knowledge, previously proposed DCNN-based grading tools lack a preliminary measure of uncertainty, which is an initial important step towards an explainable computational pathology. Developing an uncertainty quantification component can contribute to the recognition of multiple regions of ambiguity that may be clinically instructive. It also allows pathologists and medical professionals to rate images that should be prioritized for pathology annotations. Despite the existence of DCNN models and their high potential in minimizing the workload burden from pathologists, a limited number of microscopy images would require pathologists’ assistance.

In this paper, we propose a novel Entropy-based Elastic Ensemble of DCNN models (3E-Net) (The code is available at https://github.com/zakariaSenousy/3E-Net-Model (accessed on 15 May 2021)) for the automated grading of breast carcinoma using histopathological images. 3E-Net has an elasticity capability in allocating different classifiers (e.g., DCNNs) for each particular image. Our model is supported by an uncertainty quantification component which helps pathologists to refine annotations for developing more robust DCNN models that can meet their needs. Conversely, in this work, we first extract patches from the input image. Then, we designed a patch feature extractor network (i.e., pre-trained and fine-tuned DenseNet-161 [11]) to learn salient features from image patches. The extracted feature maps are then fed into multiple image-wise CNN models which are designed to capture multi-level spatial dependencies among the patches. Eventually, an uncertainty-measure ensemble-based component is introduced to select the most certain image-wise models for the final image grading. The performance of our model is evaluated on the Breast Carcinoma Histological Images dataset [12], which consists of 300 high-resolution hematoxylin-eosin (H&E) stained breast Histopathological images, divided into three invasive grades.

The contributions of this paper are summarized as follows: (1) a novel uncertainty-aware component adapted by an entropy formula to measure how confidence DCNN models of our automated breast cancer grading system on input images. This uncertainty-aware mechanism assists pathologists in identifying the complex and corrupted images which are hard to be graded by automated systems; (2) an automatic exclusion of poor histopathological images for manual investigation; (3) a new elastic ensemble mechanism is proposed using most certain DCNN models, where each input image will be classified by a pool of models, but only confident ones contribute toward the final prediction using a dynamic ensemble modeling mechanism; and (4) quantitative and qualitative analysis study have been conducted using our automated grading system on breast carcinoma dataset. To the best of our knowledge, this is the first attempt to introduce an entropy-based uncertainty quantification metric to achieve an elastic-based ensemble of DCNN models in automated grading of invasive breast carcinoma from histopathological microscopic images.

The paper is organized as follows. In Section 2, we review the related work in breast cancer grading using histopathological images. Section 3 describes the dataset used in this work. Section 4 discusses, in detail, the architecture of our proposed 3E-Net model. Section 5 describes our experimental results and discusses our findings. Section 6 concludes our work and presents future work.

## 2. Related Work

The grading process using automated feature extraction models differs from bio-markers (e.g., counting the number of cells), where the learning models depend on extracting features from the input digitized image automatically unguided by any bio-marker. The visual appearance of cells in the image is automatically processed and learned by automated models to generate prominent features. These features are then used to produce the final class label (i.e., one of the invasive carcinoma grades) in the classification problem. More precisely, automated grading is considered as a classification task that is based on the features extracted from the visual representation of the number of cells in a given image. In this section, we review the related work based on three aspects: (1) traditional handcrafted feature-based methods, (2) deep learning-based methods, and (3) ensemble-based methods.

### 2.1. Classical Handcrafted Feature-Based Methods

Several classical approaches for detecting and grading breast cancer in histological images have been introduced in the literature [13,14,15,16]. The majority of such methods concentrate on segmenting and distinguishing histological primitives such as nuclei, as well as extracting relevant features. For instance, Doyle et al. [17] proposed a method for automatically grading breast cancer histological images. Their approach combined spectral clustering with textural (including Gabor, Grey Level, and Haralick) and architectural (including Voronoi diagram, Delaunay triangulation, minimal spanning tree, and nuclear characteristics) attributes. In another work, using the log-Gabor wavelet transform and the least square support vector machine (LS-SVM) classifier, Niwas et al. [18] captured color textural features for breast cancer diagnosis. Khan et al. [19] suggested grading nuclear atypia in breast histopathological microscopy images using the geodesic geometric mean of regional co-variance descriptors as an image-level function.

Barker et al. [20] proposed a method that uses a coarse-to-fine study of pathology images’ localized characteristics. Their method has two stages. The first stage examines the range of coarse regions across the entire slide image. This involves extracting spatially localized shape, color, and texture features from tiled regions that cover the entire slide. The second stage examines a single representative tile in greater depth. Each representative tile receives a diagnostic decision value from an Elastic Net classifier. To get a diagnosis at the entire slide level, a weighted voting scheme aggregates the decision values from these tiles. The work conducted by Filipczuk et al. [21] used a circular Hough transform to identify nuclei and then used four separate classifiers to extract a series of features for biopsies classification. Zhang et al. [22] proposed a classification scheme using a one-class kernel theory component analysis model ensemble with various features derived from a grey level co-occurrence matrix. Finally, Vink et al. [23] suggested an adjusted AdaBoost algorithm to construct two nucleus detectors that concentrate on various aspects of nuclei presence for nuclei detection.

Although these conventional approaches are simple to incorporate and easy to train/ use, they are feature-dependent and computationally expensive due to (1) the use of pre-processing steps such as segmentation, nuclei separation, and detection, and (2) the lack of heuristics to guide the feature extraction.

### 2.2. Deep Learning-Based Methods

Many researchers have turned to more robust and sophisticated approaches, such as DL, to learn directly from input images. More precisely, Shaban et al. [24] proposed a colorectal cancer grading model to integrate a larger context using a context-aware neural network. To make a final prediction, this model transforms the local representation of a histology image into high-dimensional features, then combines the features by perceiving their spatial arrangement. Zhou et al. [25] introduced a new cell-graph convolutional neural network (CGC-Net) for grading of colorectal cancer, which transforms each large histology image into a graph, with each node represented by a nucleus within the input image and cellular associations denoted as edges among these nodes based on node similarity.

Sornapudi et al. [26] introduced a DL-based nuclei segmentation technique, which is based on collecting localized information through super-pixels generation using a basic linear iterative clustering algorithm and training with a CNN. Their framework detects nuclei and classifies them into one of squamous epithelium cervical intraepithelial neoplasia (CIN) grades. The work introduced by Li et al. [27] proposed a DCNN architecture for fine-grained classification and grading in breast cancer histopathological images. Their architecture has three stages. First, they integrated multi-class recognition and verification tasks of image pairs in the representation learning process. Second, a piece of prior knowledge is developed during the feature extraction process, where the variance in feature outputs between different sub-classes is significantly large while the variance within the same subclass is minimal. Finally, the feature extraction method incorporates prior knowledge that histopathological images with various magnifications belong to the same classification.

Awan et al. [28] introduced a novel metric called Best Alignment Metric (BAM) for measuring the shape of glands in colon cancer. They showed a correlation between glandular shape metric and grade of the tumor. Their model is based on a DCNN for detecting gland boundaries and a support vector machine (SVM) classifier is used for deciding the grade of cancer. Arvaniti et al. [29] presented a DL approach for automated Gleason grading of prostate cancer tissue micro-arrays with (H&E) staining. Their system was trained using detailed Gleason annotations. The work proposed in [30] developed a DL-based model for clinical-grade detection of microsatellite instability in colorectal tumors.

Recently, Munien and Viriri [31] investigated the use of the EfficientNet architecture for the classification of H&E stained breast cancer histology images. They used seven EfficientNets that are fine-tuned and tested to distinguish images into four categories: normal, benign, in situ carcinoma, and invasive carcinoma. Likewise, the work introduced by Alzubaidi [32,33] proposed a study to optimize the performance of breast cancer classification using novel transfer learning techniques. Their work suggested a transfer learning method that involved training a DL model on vast unlabeled medical image datasets and then transmitting the information to train a DL model on a limited number of labeled medical images. In addition, they built a hybrid DCNN model using a combination of ideas such as parallel convolutional layers, residual connections, and global average pooling.

Despite the success of single CNNs, several computer vision challenging problems (such as the limited availability of training images, high-level of noise and high variability of the morphological architecture of region of interests in images) still persist, multiple CNNs models are required to improve diversity to cope with complicated cases.

### 2.3. Ensemble-Based Methods

Due to the challenging problems stated earlier concerning histology images, researchers proposed the adoption of the ensemble approach. This approach is based on combining multiple DCNN models with different learning perspectives, which consequently improves diagnosis accuracy.

Yang et al. [10] proposed a CNN ensemble model called Ensemble of Multi-Scale Network (EMS-Net) to classify H&E stained breast histopathological images. EMS-Net allows to extract features using multiple pre-trained CNN models at multi-scale and select the optimal subset of the fine-tuned deep models. Kassani et al. [34] introduced an ensemble DL-based approach for automatic binary classification of breast histology images. The proposed model utilizes three pre-trained CNNs (VGG19, MobileNet, DenseNet) for feature extraction. The extracted features are then fed into a multi-layer perceptron classifier to carry out the classification task. Marami et al. [35] proposed an automated classification method for identifying micro-architecture of tissue structures in breast histology images. Their proposed architecture is based on ensembling multiple Inception networks which are trained using different data subset sampling and image perturbation. Their Inception network is modified by using adaptive pooling which increases the practical utility of their trained network, as it can be applied to images with minor scale changes from the input training images. Nguyen et al. [36] introduced a feature concatenation and ensemble method to combine several CNNs with different depths. The proposed model is made up of three pre-trained transfer learning models (Inception-v3, ResNet152, and Inception-ResNet-v2) and a fourth multi-feature-extractors model. The three feature maps collected from the three base modes are concatenated into a longer feature vector. In the end, the ensemble learning technique is used to ensemble the four feature maps (three from the base models and one from the multi-feature descriptor).

Most recently, Hameed et al. [37] introduced an ensemble model for the classification of non-carcinoma and carcinoma breast cancer histopathology images. They used different models based on pre-trained VGG16 and VGG19 architectures. Then, they followed an ensemble strategy by taking the average of predicted probabilities. Gifani et al. [38] proposed an ensemble of deep transfer learning for automated detection of COVID-19 Computed tomography (CT) scans. They used a total number of 15 pre-trained CNNs which are fine-tuned for the target task. Their ensemble method is based on the majority voting of the best combination of CNN models’ outputs. Finally, the work introduced in [39] proposed an ensemble of DCNNs for multi-class classification and textural segmentation of histopathological colorectal cancer tissues.

All the mentioned work in this subsection has shown different methods to improve the performance of diagnosis using the standard ensemble approach. However, they lack (1) the measure of confidence in the automated grading and classification, as well as, (2) the elastic ensemble of multiple DCNN models. These two components are of importance to increase the trust in the model by (1) making sure that only models with a pre-defined degree of confidence contribute to the prediction, and by (2) flagging out cases that are hard to classify confidently by the model for further inspection.

## 3. Dataset

**Figure 1:**
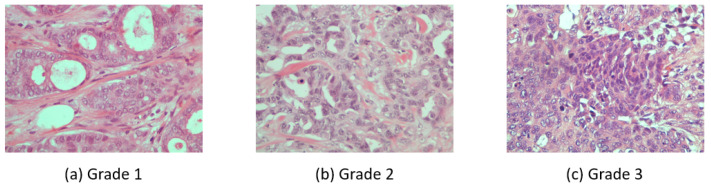
Three H&E stained breast histopathological microscopy images from different invasive carcinoma grades.

Breast carcinoma histological images [12] were used for this work. The dataset contains cases of breast carcinoma histological specimens collected in the department of pathology, “Agios Pavlos” General Hospital of Thessaloniki, Greece. The dataset is composed of 300 H&E stained breast histopathological microscopy sections with the size of 1280 × 960 pixels. The dataset is mainly categorized into three grades of invasive carcinoma: grade 1, grade 2, and grade 3 (See Figure 1).

The categories are divided as 107 images for grade 1, 102 images for grade 2, and 91 images for grade 3. These images are associated with 21 different patients with invasive ductal carcinoma of the breast. The image frames are from tumor regions taken by a Nikon digital camera connected to a compound microscope with a 40× magnification objective lens.

## 4. Proposed 3E-Net Model

**Figure 2:**
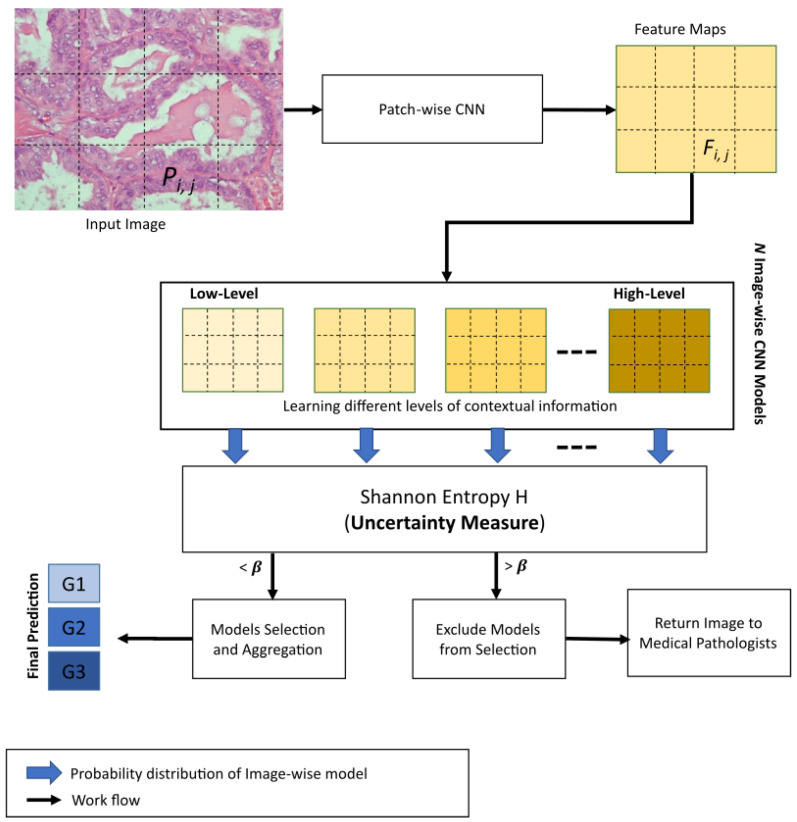
Overview of 3E-Net. The model starts by taking a histopathological image section as input. Several small patches are extracted from the image where $P_{i,j}$ is one of the extracted patches. All patches are then fed into a patch-wise CNN for feature extraction, where $F_{i,j}$ is one of the extracted feature maps. Feature maps are then inserted into *N* image-wise CNN models to learn multiple levels of spatial dependencies information. Finally, Shannon entropy *H* is adopted in our uncertainty-aware component to measure the sensitivity of the input image to the *N* image-wise models. According to a pre-defined threshold β, the most certain models were selected for final grading prediction. In case of having zero certain models, the input image is returned to medical professionals for manual exploration and further investigation.

In this section, we describe, in detail, our proposed 3E-Net model. Given a histopathological image section with a high resolution (1280 × 960 pixels) as an input, the main target is to grade the image into one of three invasive grades of breast cancer: grade 1, grade 2, or grade 3. As illustrated by Figure 2, our model consists of several DCNNs which are designed and implemented based on the input size of the image and the number of patches extracted from the image. First, the input image is divided into many smaller patches which are then inserted into a pre-trained and fine-tuned DCNN which acts as patch-wise feature extractor network. Second, the extracted feature maps are fed into image-wise networks which encode different levels of contextual information. As a final and prominent step, the final image predictions (i.e., grades) from image-wise models are then inserted into an elastic ensemble stage which is mainly based on measuring the uncertainty of predictions in each model. This uncertainty measure of predictions is designed using the Shannon entropy [40] which measures the level of randomness in the model’s final prediction. More precisely, Shannon entropy values of different models in our ensemble architecture were used to select the most accurate/certain models (i.e., the models which have a small entropy value) to improve the elasticity capability of 3E-Net in allocating different classifiers and improving diversity. Using a pre-defined threshold, only models with a high degree of certainty are included in the final elastic ensemble of the image.

### 4.1. Patch-Wise Feature Extraction

Due to the scarcity of annotated training data in the medical field, transfer learning [41] has emerged as a prominent approach to cope with the problem. Transfer learning is a mechanism that uses machine learning models (e.g., CNNs) which are pre-trained on large datasets (e.g., large-scale images of ImageNet dataset) to be adapted and used in different domain-specific tasks (e.g., breast cancer grading). In such mechanisms, the network configuration is preserved, and the pre-trained weights are used to configure the network for the new domain-specific task. During the fine-tuning stage, the initialized weights are continuously updated, allowing the network to learn hierarchical features relevant to the desired task. Fine-tuning is effective and robust for various tasks in the medical domain [8,10,42].

As stated earlier, the patch-based paradigm proved to be effective when it comes to high resolution histopathological images [7,8,10,42]. In this work, we utilize a pre-trained and fine-tuned DenseNet-161 to act as feature extractor networks for image patches. DenseNet-161 has demonstrated a superb performance for ILSVRC ImageNet classification task [43]. Moreover, DenseNet-161 has shown a great success in several histopathological image analysis pipelines [10,44,45,46,47,48,49,50,51]. In order to supply the patch-wise feature extractor network with image patches, we extract a number of patches *k* based on the following equation [7]:

(1) $$k=\left(1+\left\lfloor \frac{W-w}{s}\right\rfloor \right)\times \left(1+\left\lfloor \frac{H-h}{s}\right\rfloor \right)$$

where *W* and *H* are width and height dimensions of the input image, respectively. While, *w* and *h* are width and height dimensions for the image patch, respectively and *s* is the stride used over the input image.

To improve variety (in the training data) and alleviate overfitting for the patch-wise feature extractor network, we extracted and used partially overlapped patches. Furthermore, we applied data augmentation techniques by transforming each patch using rotation and reflection operations. For example, random color alterations introduced by [52] has been applied to each patch as it aids in minimizing the visual diversity of the patches. Our model learns rotation, reflection, color invariant characteristics, and makes pre-processing color normalization [53]. The patch-wise feature extractor network is then trained using categorical cross-entropy loss based on image-wise labels. The loss equation is defined as:

(2) $$L\left(y-\hat{y}\right)=-\sum_{i=1}^{c}y_{i}\log{\hat{y}}_{i}$$

where $y_{i}$ and ${\hat{y}}_{i}$ represent the ground truth label and the prediction of each class *i* in *c* classes, respectively.

### 4.2. Image-Wise Grading

Once the feature extraction is accomplished, feature maps are fed into multiple image-wise networks to encode multi-level contextual information. The main purpose of the image-wise network is to grade images based on local and contextual features captured from image and spatial dependencies information between different patches, respectively.

During the training stage of an image-wise network, we extract non-overlapping patches from the input image, where they are used to form newly concatenated feature maps that are designed based on neighboring feature maps only. This criterion helps in building the intended contextual information. In our model, we build various image-wise networks that are based on multi-levels of contextual information. Each patch in the image has its own feature map. The number of image-wise network models depends on the number of feature maps extracted from the image and the possible formed shapes of neighbor feature maps. The contextual levels have low-level context which builds contextual feature maps among 2 original neighboring feature maps only, and high-level context builds contextual feature maps among all the original feature maps extracted from the image. For instance, having *q* feature maps extracted from the input image helps in generating image-wise models which learn contextual information among 2 feature maps (low-level) to *q* feature maps (high-level). Furthermore, for each level of contextual information (except for the highest level), a number of image-wise models can be generated based on different shapes of the neighbor feature maps. The formation and concatenation of any two or more feature maps can have different shapes. Likewise in the patch-wise network, the data augmentation process is applied to dataset images by applying rotation, reflection, and color alterations. In addition, categorical cross-entropy loss is used in the training process against the corresponding image-level labels.

Image-wise CNN is composed of a series of 3 × 3 convolutional layers followed by a 2 × 2 convolution with a stride of 2 for down-sampling. Batch normalization and ReLU activation function were attached after each layer. A 1 × 1 convolutional layer is used before the classifier to obtain the spatial average of feature maps. As a final block, the network ends with 3 fully connected layers and a log softmax classifier. The softmax activation function is defined as:

(3) $$S\left(z_{i}\right)=\frac{e^{z_{i}}}{\sum_{j}^{c}e^{z_{j}}}$$

where $z_{i}$ represents output element *i* of the last fully connected layer.

### 4.3. Elastic Ensemble Using Uncertainty Quantification

In this section, we describe our elastic ensemble of the constructed image-wise models. As a crucial step in this work, we transform the standard ensemble-based model into an elastic ensemble model which dynamically selects models based on the uncertainty of models as a measuring factor. In other words, for each image, a dynamic number of models is selected and combined towards the final image prediction. To measure uncertainty for our ensemble model, we adopted Shannon entropy for each image-wise model. The formula for Shannon entropy is represented as:

(4) $$H\left(X\right)=H\left(p_{1},\ldots ,p_{c}\right)=-\sum_{i=1}^{c}p_{i}\log_{2}p_{i}$$

where H(X) represents Shannon entropy for input image *X* and $p_{1},\ldots ,p_{c}$ is probability distribution for image *X* on *c* class categories.

During the testing stage, the input image is graded using all the image-wise models in an ensemble-based model. Each model generates the grading of the image in the form of a probability distribution for *c* class categories. Then, these probability distributions are evaluated using Shannon entropy (based on an uncertainty threshold value (β)) to measure uncertainty. According to the calculated uncertainty measure, a dynamic number of image-wise models will be selected for each image.

The selection process of image-wise models in the elastic ensemble process works by comparing the Shannon entropy measure evaluated for a particular model against a pre-defined threshold value β, as defined in the experimental study. If the entropy value is less than β, then the model will be chosen and included in a list of chosen models for a particular image. In the end, each image in the dataset should have a dynamic number of chosen models to produce the final prediction. In case of having images with zero chosen models, we prioritize these images for pathology annotating by medical professionals. After selecting the most certain image-wise models, the class predictions of these models are aggregated to produce the final class prediction distribution.

Algorithm 1 provides a detailed description of 3E-Net model. The input image is divided into smaller patches. Then, using patch-wise CNN, many feature maps are extracted. These feature maps are then inserted into image-wise CNN models. Each image-wise model produces a probability distribution of the input image. In the end, the Uncertainty-aware component is utilized to measure the level of uncertainty for each image-wise model’s prediction. The models with uncertainty values less than a threshold β are chosen and their predictions are aggregated for final grading $\hat{y}$. If the input image has no chosen models, medical professionals are involved in the final grading decision.
**Algorithm 1:** 3E-Net Model
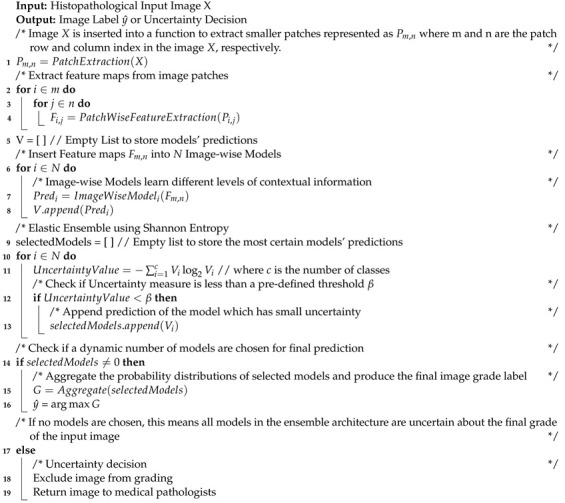


## 5. Experimental Study

We evaluated the performance of our work on the Invasive Breast Carcinoma dataset. As aforementioned, the dataset has 300 images which all are used for training the ensemble model using 5-fold cross-validation. Cross-validation enables us to overcome the limited availability of annotated images, making sure that the model is well-trained. For training patch-wise networks, we used microscopy patches extracted from training images. These patches are augmented using rotation, flipping, and colorization methods. Similarly, in image-wise networks, the same training process is conducted, but using the image-level dataset instead of patches. In the experimental study, we designed and implemented two standard ensemble models. First, the baseline ensemble model which has DenseNet-161 as the patch-wise feature extractor CNN will be denoted by Standard Ensemble Model (Version A). Second, we applied a modification by using the patch-wise CNN introduced in [7] as the feature extractor of the ensemble model. The modified ensemble model will be denoted by Standard Ensemble Model (Version B). Finally, our contribution has two 3E-Net models: 3E-Net Version A & 3E-Net Version B, where we apply elastic ensemble approach to the standard ensemble models.

### 5.1. Hyperparameter Settings

As we have DenseNet-161 as the patch-wise feature extractor of the baseline ensemble model (Standard Ensemble Model (Version A)), we extracted patches of size 224 × 224 from the input image. Consequently, a number of 20 non-overlapped patches can be generated (where the original size of the input image is 1280 × 960) to extract high-level contextual information. However, due to the limited GPU memory, we down-sampled the input images to a smaller scale of 896 × 672.

For training data extraction, we set the stride to s = 112 to extract partially overlapped patches for both versions (A & B). This stride value helps in increasing the training patch samples for patch-wise CNN and prevents the network from overfitting. We applied data augmentation by rotating the training patches by 90 degrees with horizontal and vertical flipping. To fine-tune the patch-wise CNN for Standard Ensemble Model (Version A) to our grading task, we modified the number of output neurons from 1000 to only 3 (as we have three grades). We used Adam optimizer [54] for minimizing the cost function and we set the learning rate to 0.0001 for 5 training epochs and batch size to 32 for both patch-wise CNNs in versions A & B.

The extracted feature maps from patch-wise CNN are then inserted into image-wise models. For training image-wise model, we extracted non-overlapped patches from the new image scale giving us 12 patches by using s = 224. This means that we have a total number of 12 feature maps represented as a matrix of size (3×4) (as shown in Figure 2) to be used for the training process of image-wise models. Different levels of contextual information have been learned by combining all the original feature maps to form multi-level contextual feature maps. For example, the lowest-level contextual feature maps are generated by combining 2 neighboring feature maps while the highest-level contextual feature maps are generated by combining the 12 feature maps of the image. As mentioned earlier, different shapes of neighbor feature maps can be generated from each contextual level (except for the high-level as we combine all the 12 feature maps). Once the different levels of contextual feature maps are constructed, a number of DCNNs will be set up to learn the multi-level contextual information. This results in an arbitrarily chosen number of 17 image-wise models to form our ensemble architecture. Image-wise CNNs are trained on augmented image-level samples by applying rotation of 180 degrees with flipping. The remaining settings are the same as patch-wise CNN except that each image-wise CNN is trained for 10 training epochs and a batch size of 8.

Finally, we design and implement an elastic ensemble approach (3E-Net Versions A & B) for the standard ensemble models. This is accomplished using Shannon entropy to measure the uncertainty of the 17 image-wise models. Each input image can have a dynamic number of models less than 17 based on the pre-defined β which excludes the models with high uncertainty values. We used a wide range of β values from $10^{-8}$ to 2 to demonstrate the capability of 3E-Net versions to provide high performance.

### 5.2. Quantitative Evaluation

We adopted accuracy, precision, recall, and F1-score metrics to evaluate the performance of our model. Precision is the classifier’s capability to not mark a result as positive if it is negative, the classifier’s recall is its ability to locate all positive samples, and F1-score can be expressed as the harmonic mean of the precision and recall. The accuracy, precision, recall, and F1-score were determined as follows:

(5) $$Accuracy=\frac{TP+TN}{TP+TN+FP+FN}$$

(6) $$Precision=\frac{TP}{TP+FP}$$

(7) $$Recall=\frac{TP}{TP+FN}$$

(8) $$F1-\mathrm{score}=2\cdot \frac{Precision\times Recall}{Precision+Recall}$$

where TP and TN represent the correct predictions by our elastic ensemble models for the occurrence of a certain grade or not, respectively, while FP and FN are the incorrect model predictions for all cases.

#### 5.2.1. Performance of Standard Ensemble-Based Models

Table 1 and Table 2 illustrate precision, recall, F1-score and grading accuracy of standard ensemble of DCNNs (i.e., ensemble of the total 17 models) for Version A and Version B, respectively. Table 1 and Table 2 show that both ensemble models can effectively differentiate grade 2 from the two other grades (grade 1 and grade 3). Moreover, Version A and Version B have achieved an average precision of 93.04% and 90.98%, respectively, while they achieved average grading accuracy of 93% and 90.68%, respectively.

#### 5.2.2. Performance of 3E-Net Models

To evaluate the performance of the uncertainty-aware component, we further investigate the grading accuracy of the elastic ensemble approach. Moreover, for a fair comparison with the standard ensemble-based models, we introduced two new metrics: (1) Weighted Average Accuracy (WAA), which measures the average of grading accuracies for the 5 folds in the dataset weighted by the number of the included images in each fold; and (2) Abstain percentage (AP): measures the percentage of the excluded images to the total number of images in the dataset. The formulation of the two metrics are determined as follows:

(9) $$WAA=\frac{1}{\sum_{i=1}^{t}d_{i}}\sum_{i=1}^{t}Accuracy_{i}*d_{i}$$

(10) $$AP=\left(\frac{\sum_{i=1}^{t}R_{i}}{DS}\right)\times 100$$

where $d_{i}$ and $Accuracy_{i}$ represent the number of included images and grading accuracy in fold *i* over a total number of *t* folds, respectively, $R_{i}$ is the count of the excluded images in fold *i*, and DS is the total number of images in the dataset

Table 3 demonstrates the capability of our elastic ensemble approach in providing higher grading accuracies for both 3E-Net model variations (Version A & B) when compared to the standard ensemble models. Moreover, such improvement in the grading accuracies indicates that the excluded images are difficult to classify by the DCNN models, where a manual investigation is required for such images. It can be noticed that 3E-Net models achieve the highest accuracies of 96.15% ($\beta =5\times 10^{-7}$) and 99.50% ($\beta =5\times 10^{-6}$) for Version A and Version B, respectively. As illustrated by Table 3, the other threshold β values yield grading accuracy of ∼95% for Version A and ∼99.40% for Version B.

**Figure 3:**
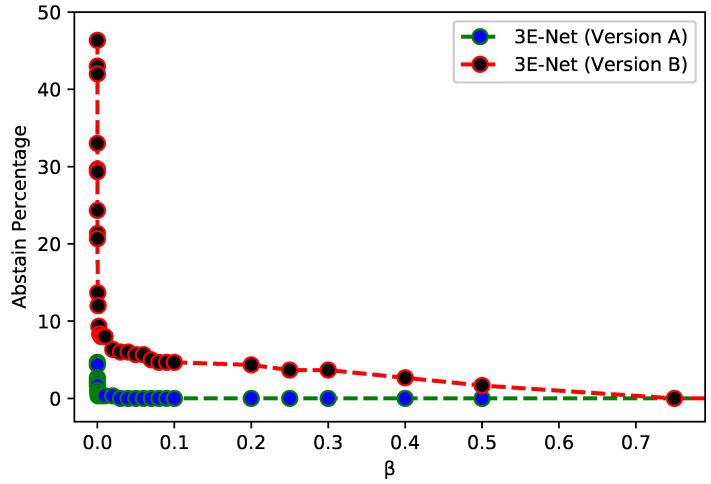
AP of excluded images for 3E-Net Version A (Blue) and 3E-Net Version B (red) over a range of threshold β values using elastic ensemble on Invasive Breast Carcinoma Dataset.

**Figure 4:**
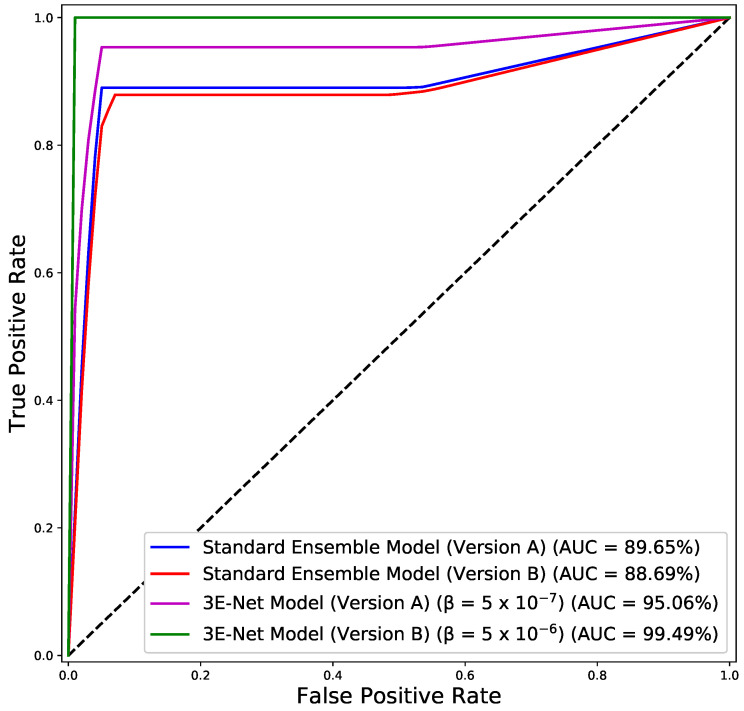
ROC curves for the standard and elastic versions of our models (A & B).

**Figure 5:**
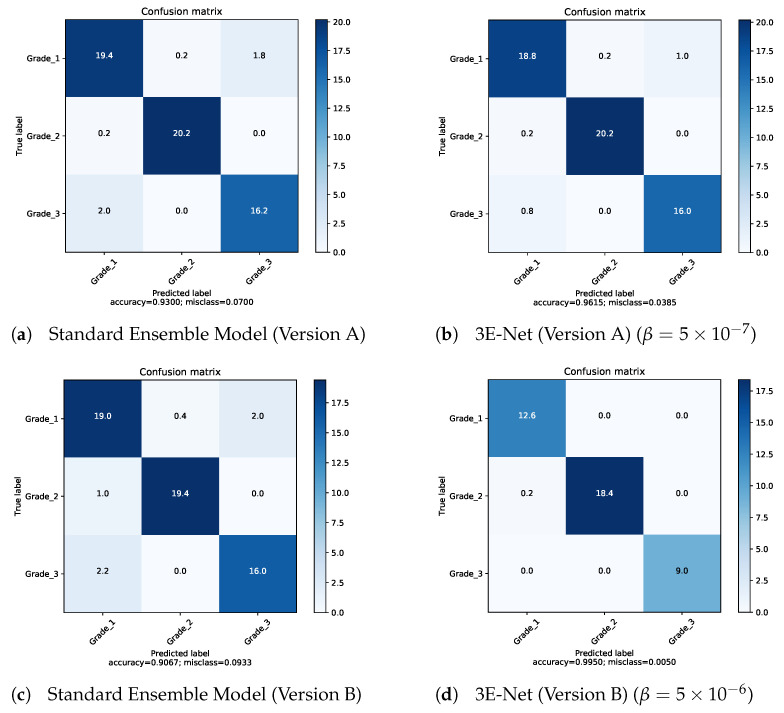
Confusion matrices for our proposed models.

Figure 3 depicts AP of the excluded images from the dataset over different values of β for 3E-Net models (Version A & Version B). The curves show that AP decreases when we increase β. In addition, starting from β=0.75, the number of excluded images reaches zero for both models. Figure 4 depicts the ROC curves for both model versions using the standard and elastic ensemble-based approaches, see also Figure 5 for the confusion matrices obtained by our models.

**Figure 6:**
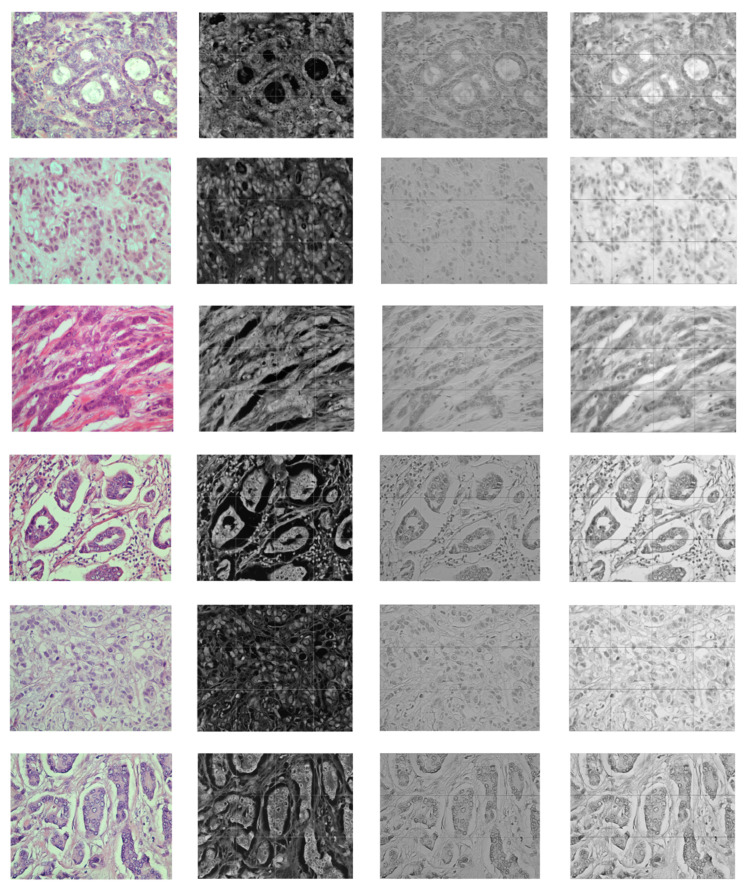
Examples of feature maps obtained by multiple filters learned within the first convolutional layer of the patch-wise network of standard ensemble (version B). The colored image is the original, while the gray-scale images are the output maps.

**Figure 7:**
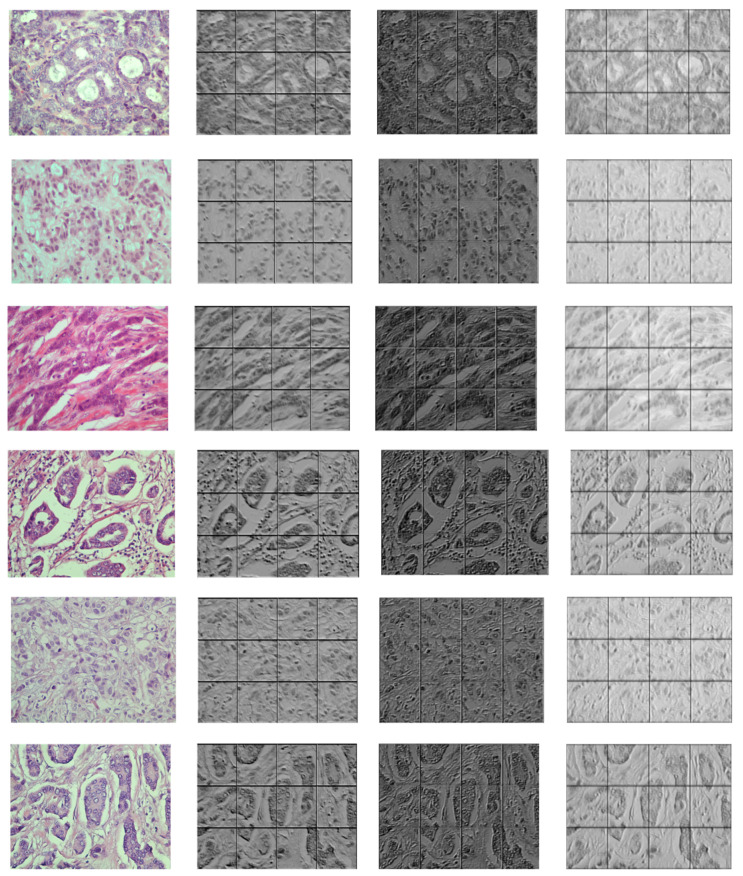
Examples of feature maps obtained by multiple filters learned within the last convolutional layer of the patch-wise network of standard ensemble (version B). The colored image is the original, while the gray-scale images are the output maps.

Figure 6 and Figure 7 demonstrate the output visualizations of multiple filters applied to the first and last convolutional layers of the patch-wise network of the standard ensemble model (version B). Note how the feature maps are distinctive in terms of their morphological structures.

#### 5.2.3. Comparison with Different Methods

To demonstrate the effectiveness of our solution, we applied ablation study by comparing the performance of a state-of-the-art single DCNN model, standard ensemble-based models, and our elastic ensemble approach. In Table 4, we compare our 3E-Net models with the state-of-the-art models in digital breast pathology, namely DCNN+SVM model [8], deep spatial fusion CNN model [9], two-stage CNN model [7], and ensemble of multi-scale networks (EMS-Net) [10]. As demonstrated by Table 4, our 3E-Net model outperformed both the recent models in the literature and the standard ensemble models.

#### 5.2.4. Performance of 3E-Net on BreakHis Dataset

To confirm the effectiveness of 3E-Net model, we applied 3E-Net model (version A) on the Breast Cancer Histopathological Database (BreakHis) [55]. BreakHis has a total number of 7909 breast cancer histopathology images taken from 82 patients using different magnifying factors (40×, 100×, 200×, and 400×). The dataset is divided into 2480 benign and 5429 malignant microscopic images with a resolution of 700 × 460 pixels. We use 40× magnification images which has 625 benign and 1370 malignant samples.

Here, we down-sampled the images to around 80% of the original scale (448 × 336). This image scale produces 6 image-wise CNNs to be used in the ensemble process. We also used the same hyperparameter settings except for patch-stride values, where we used s = 28 for training the backbone network (DenseNet-161) and s = 112 for training the 6 image-wise CNNs. Finally, as the BreakHis dataset contains only two classes (benign or malignant), we fine-tuned DenseNet-161 by updating the number of neurons from 1000 to only 2 neurons in the last fully connected layer. As shown in Table 5, our model has proved to be effective on both standard and elastic ensemble. We applied 5-fold cross validation and achieved a classification accuracy of 99.80% using standard ensemble technique. In addition, the results show the validity of our novel elastic method of 3E-Net on different beta values by improving the performance, where an accuracy of 99.95% has been achieved on (β = $9\times 10^{-6}$).

### 5.3. Qualitative Evaluation

**Figure 8:**
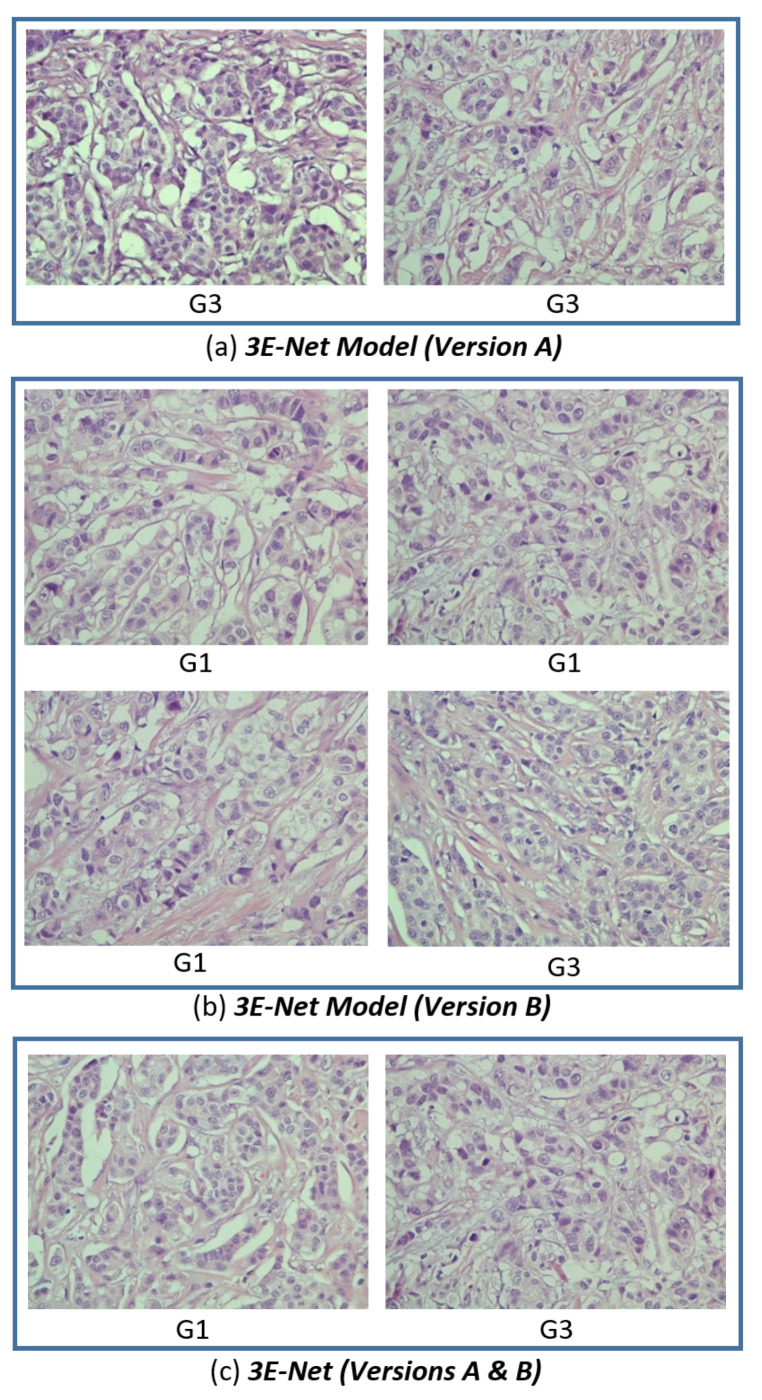
Highly uncertain excluded images from the grading process of our dynamic ensemble-based models. The excluded images come from three perspectives: (**a**) 3E-Net Model (Version A), (**b**) 3E-Net Model (Version B), and (**c**) Versions A & B combined. Each image in the figure has a caption that presents the ground truth label (G1: grade 1 and G3: grade 3).

To quantitatively evaluate the performance of our model on the excluded images, we set β to a high value to find images that are less sensitive and highly uncertain to the 17 image-wise models in the ensemble of DCNN models. Figure 8 shows the images, for which all the image-wise models in the ensemble agree on the uncertainty decision based on the high uncertainty values resulted from these models. Figure 8c shows two images from the selected excluded images which are agreed on their uncertainty by both 3E-Net model variations (Version A and Version B). Moreover, it can be noticed that the highly uncertain images come from grade 1 or grade 3, which proves trustworthy of our results in Table 1 and Table 2 to show how it is slightly hard to differentiate between grade 1 and grade 3.

Based on the sample of the excluded images shown in Figure 8, we returned to a domain expert to further investigate the possible reason behind the high uncertainty of the excluded images. The uncertainty may be due to usage of datasets from heterogeneous populations [56], or reduced sample size used in the study [57]. In this regard, additional information depending on the staining of specific biomarkers for breast cancer grading such as Ki67 [58] could be used to resolve the diagnostic uncertainty in CNN.

## 6. Conclusions and Future Work

In this paper, we proposed 3E-Net model to grade invasive breast carcinoma using histopathological images into three grades: grade 1, grade 2 and grade 3. Our model has the capability to learn multi-levels of contextual information using image patches through various image-wise CNN models. Moreover, our ensemble model has been designed in a way to measure the level of randomness (using a novel entropy-based formula) in the input images and quantify the challenges in grading images. We evaluated our proposed grading system on Invasive Breast Carcinoma Dataset from ‘Agios Pavlos’ General Hospital of Thessaloniki, Greece. Our elastic ensemble model has two variations that achieved grading accuracy of 96.15% and 99.50% in the five-fold cross-validation on training images and outperformed standard ensemble-based models and a state-of-the-art method. 3E-Net proved its effectiveness in excluding the uncertain microscopy images to be investigated and explored by medical professionals.

As a future development, our work can be extended by introducing different patch-wise CNNs and applying different learning perspectives while building the ensemble of DCNN models. This is by learning and integrating different kinds of features including global, local, and contextual information to improve the robustness and diversity of the ensemble model. Moreover, our solution can be adapted to other applications (e.g., diagnosis) and cope with different histopathological tissues such as prostate and colorectal cancer.

## Figures and Tables

**Table 1:** Grading performance (mean) of standard ensemble model (**Version A**) on Invasive Breast Carcinoma dataset using 5-fold cross validation.

Grade	Precision	Recall	F1-Score	Accuracy
Grade 1	89.86%	90.65%	90.25%	93.00%
Grade 2	99.05%	99.05%	99.02%	99.33%
Grade 3	90.05%	89.00%	89.51%	93.67%
Total	93.04%	93.00%	93.01%	93.00%

**Table 2:** Grading performance (mean) of standard ensemble model (**Version B**) on Invasive Breast Carcinoma dataset using 5-fold cross validation.

Grade	Precision	Recall	F1-Score	Accuracy
Grade 1	85.83%	88.83%	87.21%	90.68%
Grade 2	98.09%	95.14%	96.48%	97.68%
Grade 3	89.04%	87.89%	88.39%	93.00%
Total	90.98%	90.68%	90.72%	90.68%

**Table 3:** WAA of 3E-Net Model variations (Version A & Version B) on different β values.

Model	β	Accuracy
3E-Net (Version A)	$5\times 10^{-7}$	**96.15%**
	$9\times 10^{-7}$	95.82%
	$5\times 10^{-6}$	94.86%
	$10^{-5}$	94.56 %
3E-Net (Version B)	$5\times 10^{-6}$	**99.50%**
	$10^{-6}$	99.43%
	$9\times 10^{-7}$	99.42%
	$5\times 10^{-7}$	99.38 %

**Table 4:** Comparison between different methods on Invasive Breast Carcinoma Dataset using 5 fold cross-validation.

Method	Precision	Recall	F1-Score	Accuracy
DCNN + SVM [8]	87.64%	87.38%	87.38%	87.38%
Deep Spatial Fusion CNN [9]	92.67%	92.65%	92.62%	92.65%
Two-stage CNN [7]	93.07%	92.69%	92.70%	92.69%
EMS-Net [10]	93.04%	93.00%	93.00%	93.00%
Standard Ensemble Model (Version A)	93.04%	93.00%	93.01%	93.00%
Standard Ensemble Model (Version B)	90.98%	90.68%	90.72%	90.68%
3E-Net (Version A) ($\beta =5\times 10^{-7}$)	96.23%	96.15%	96.16%	96.15%
3E-Net (Version B) ($\beta =5\times 10^{-6}$)	99.54%	99.50%	99.50%	99.50%

**Table 5:** Performance (mean) of standard and elastic ensemble models (**Version A**) on BreakHis dataset using 5-fold cross validation.

Model	β	Accuracy
Standard Ensemble Model	NA	**99.80%**
3E-Net Model	$9\times 10^{-6}$	**99.95%**
	$5\times 10^{-4}$	99.90%
	$3\times 10^{-2}$	99.85%

## Data Availability

The data presented in this study are available in [12,55].
